# Muscle spindles of the multifidus muscle undergo structural change after intervertebral disc degeneration

**DOI:** 10.1007/s00586-022-07235-6

**Published:** 2022-05-27

**Authors:** Gregory James, Carla Stecco, Linda Blomster, Leanne Hall, Annina B. Schmid, Cindy C. Shu, Christopher B. Little, James Melrose, Paul W. Hodges

**Affiliations:** 1 Centre of Clinical Research Excellence in Spinal Pain, Injury and Health, School of Health & Rehabilitation Sciences, University of Queensland, Brisbane, QLD 4072, Australia; 2 Human Anatomy and Movement Science, University of Padua, Padua, Italy; 3 Nuffield Department of Clinical Neurosciences, Oxford University, Oxford, UK; 4 Raymond Purves Bone and Joint Research Laboratories, Kolling Institute of Medical Research, Institute of Bone and Joint Research, The Royal North Shore Hospital, University of Sydney, St Leonards, NSW, Australia; 5 Graduate School of Biomedical Engineering, University of New South Wales, Sydney, Australia; 6 Centre of Clinical Research Excellence in Spinal Pain, Injury and Health, School of Health and Rehabilitation Sciences, The University of Queensland, Brisbane, QLD 4072, Australia

**Keywords:** Multifidus, Muscle spindle, Connective tissue, Intervertebral disc degeneration, Proprioception

## Abstract

**Purpose:**

Proprioceptive deficits are common in low back pain. The multifidus muscle undergoes substantial structural change after back injury, but whether muscle spindles are affected is unclear. This study investigated whether muscle spindles of the multifidus muscle are changed by intervertebral disc (IVD) degeneration in a large animal model.

**Methods:**

IVD degeneration was induced by partial thickness annulus fibrosus lesion to the L3-4 IVD in nine sheep. Multifidus muscle tissue at L4 was harvested at six months after lesion, and from six age-/sex-matched naïve control animals. Muscle spindles were identified in Van Gieson’s-stained sections by morphology. The number, location and cross-sectional area (CSA) of spindles, the number, type and CSA of intrafusal fibers, and thickness of the spindle capsule were measured. Immunofluorescence assays examined Collagen I and III expression.

**Results:**

Multifidus muscle spindles were located centrally in the muscle and generally near connective tissue. There were no differences in the number or location of muscle spindles after IVD degeneration and only changes in the CSA of nuclear chain fibers. The thickness of connective tissue surrounding the muscle spindle was increased as was the expression of Collagen I and III.

**Conclusion:**

Changes to the connective tissue and collagen expression of the muscle spindle capsule are likely to impact their mechanical properties. Changes in capsule stiffness may impact the transmission of length change to muscle spindles and thus transduction of sensory information. This change in muscle spindle structure may explain some of the proprioceptive deficits identified with low back pain.

## Introduction

Low back pain (LBP) is the foremost cause of disability [1]. LBP and/or injury induces structural [2–7] and functional [8] changes to the paraspinal muscles, that depend on the stage of chronicity. Changes in the structure of the multifidus are likely to contribute to altered muscle function and vice versa [9] and provide a plausible link to chronic LBP. Muscle spindles provide a vital contribution to proprioception, the sense of body position and movement, and are necessary for control of movement and for health of the musculoskeletal system [10] including the spine [11]. The high density of muscle spindles in the multifidus has been interpreted to suggest a critical sensory role for this muscle [12]. Proprioception is impaired in LBP (e.g., reduced positional sense and difficulty in repositioning [13]), and likely underpins sub-optimal control of spinal motion [14] with potential for a causal relationship to injury [10, 15]. Although spindles have structural similarities to extrafusal muscle (i.e., muscle excluding muscle spindles), whether they undergo similar structural change in response to LBP and injury has not been investigated.

Muscle spindles modify their discharge in response to changes in length or speed of length change in skeletal muscle [10]. They consist of a connective tissue-covered central fluid capsule in the equatorial region of the spindle and two polar regions that extend parallel to the extrafusal muscle fibers [10]. The capsule includes specialized muscle fibers (intrafusal fibers) that have non-contractile mid-regions where sensory nerve endings of primary and secondary afferents terminate. There are two distinct subtypes of intrafusal fibers, nuclear chain and nuclear bag fibers. Nuclear chain fibers have a smaller diameter and are short (restricted to the equatorial region). Nuclear bag fibers have larger diameter, include an expanded mid region, and extend beyond the capsule to the poles of the spindle.

Muscle spindle structure changes with ageing and some musculoskeletal disorders such as muscular dystrophy. In both cases, the connective tissue equatorial capsule is thickened [10], but evidence of changes to intrafusal fibers is conflicting. In animal models of muscular dystrophy, intrafusal muscle fibers are unaffected, despite substantial extrafusal muscle fiber atrophy. In humans studies of ageing, spindles include fewer intrafusal muscle fibers and show signs of denervation [10]. Changes to connective tissue, even without changes to intrafusal fibers, might affect spindle function secondary to changes in mechanics, with potential contribution to the compromised proprioception reported in both conditions.

Altered mechanics of the spindle could plausibly impact the potential for spindles to transduce information regarding muscle length change and might explain the proprioceptive deficits frequently reported in individuals with LBP [13, 15, 16]. Extrafusal multifidus muscle undergoes substantial structural change (fibrosis, change in collagen subtypes, fatty infiltration, atrophy and fiber-type change of the extrafusal fibers) as a result of neural and immune mechanisms [9]. These changes are induced by spine injury, even when the muscle is uninjured. Although direct injury to spindles and other mechanoreceptors in conjunction with spine injury has been suggested to cause proprioceptive deficits [17], the potential for uninjured muscle spindles to undergo similar structural adaption to the extrafusal fibers has not been investigated in LBP. This proposal is strengthened by the presence of changes in structure of muscle spindles (and associated proprioceptive deficits) in aging muscular and dystrophy [10].

Using an established intervertebral disc (IVD) degeneration model, this study aimed to test the hypotheses that IVD degeneration would: 1) increase the thickness of connective tissue surrounding the muscle spindles capsule and, 2) alter the collagen composition of the capsule. Similar to observations for muscular dystrophy, we predicted that IVD injury would not alter the number or location of the spindles in the multifidus muscle or induce changes to the number or cross-sectional area (CSA) of the intrafusal muscle fibers.

## Materials and methods

### Animals

The animals in this study were utilized in a parallel study examining IVD degeneration [18]. For the present study, tissue was harvested from fifteen merino sheep (aged 3–4 years) that had or had not been exposed to an IVD lesion, but no other treatment. Tissue from these animals has been used for previous experiments [2, 19–21]. Institutional ethical approval was obtained for all procedures and experiments performed in this study.

### Surgical procedure and tissue harvesting

Nine sheep (injured group) were assigned to the ‘injury group’. These animals received a single 6 × 20 mm lesion on the L1-2, L3-4 and L5-6 IVDs, as previously described [19]. This lesion induces progressive degeneration of the injured IVDs in a manner that replicates the molecular components of human IVD degeneration [22]. Post-surgery, the animals were housed individually and closely monitored to ensure their health and well-being. The animals were then housed in open paddocks with 6 animals assigned to the ‘control group,’ that did not undergo any surgical procedures. Six months post-surgery, the animals were euthanized and the multifidus muscle adjacent to the L4 spinous process was harvested. The tissue underwent fixation in 4% paraformaldehyde and dehydration before being embedded in paraffin. Transverse tissue Sects. (7 μm) were obtained and used for all experimental procedures.

### Van Gieson’s stain and analysis

**Fig. 1:**
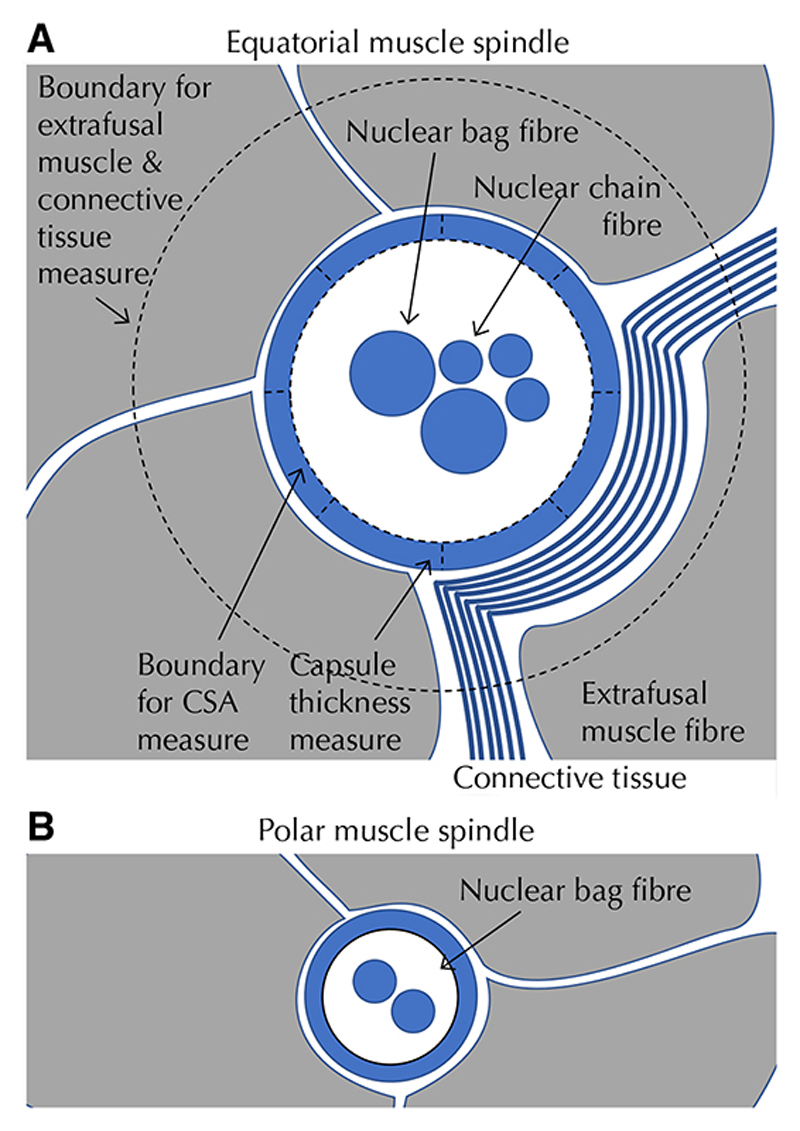
Methods for quantification of muscle spindle parameters. **a** shows features of an equatorial spindle and associated measures of spindle cross-sectional area (CSA) and capsule thickness at eight equidistant locations. The boundary used for quantification of surrounding muscle and connective tissue (two times CSA of muscle spindle is shown. **b** shows features of a polar spindle with no nuclear chain fibers and narrowed ends of nuclear bag fibers

**Fig. 2:**
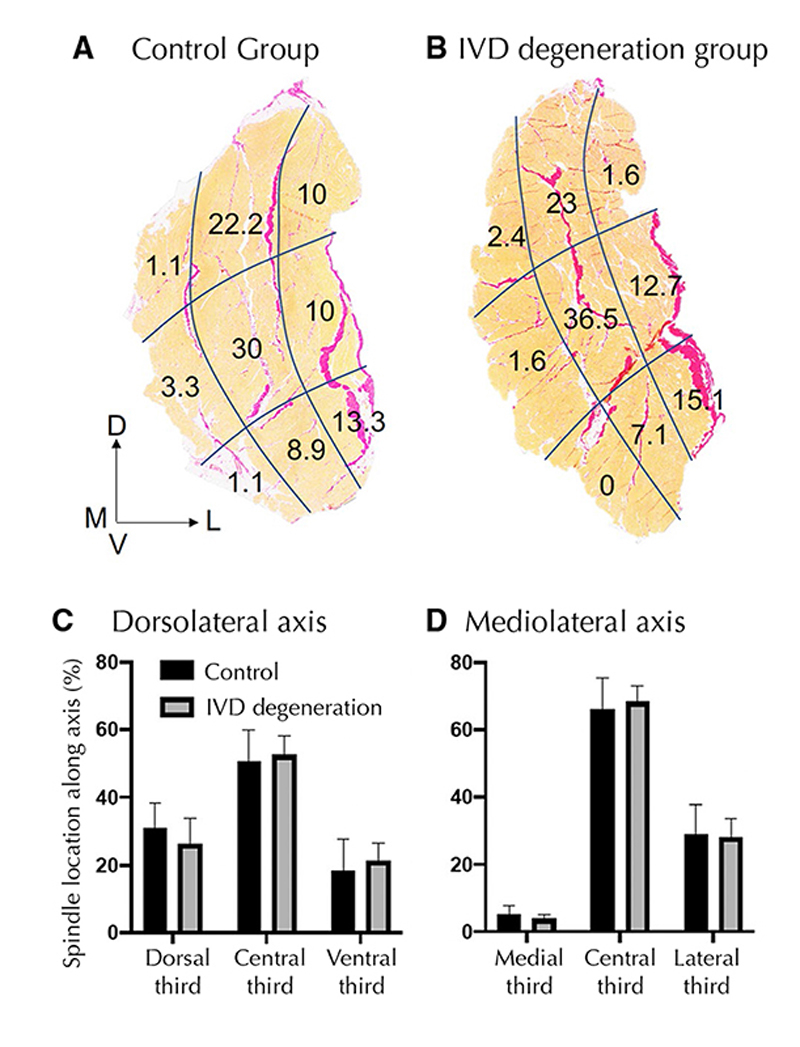
Location of muscle spindles. The multifidus muscle was divided into 3 regions in the dorsolateral and 3 regions in the mediolateral dimension. **a** and **b** show the proportion of muscle spindles in each of the regions for the control and IVD degeneration groups in sections with Van Gieson’s staining. **c** and **d** show the proportions in each third in the dorsolateral and mediolateral axes. There was no difference between groups in any location measure. D—dorsal; V—ventral; M—medial; L—lateral; IVD—intervertebral disc

Slides underwent three 2-min xylene and ethanol washes to remove wax and rehydrate the sections. The slides were then bathed in running water (2 min), Weigert’s haematoxylin (10 min) and Van Gieson’s solution (1 min). The stained sections were dehydrated, mounted and imaged (Imagescope, Leica). All muscle sections were closely examined to identify the total number and location of all muscle spindles. Spindles were identified by the presence of intrafusal muscle fibers surrounded by a connective tissue capsule (Fig. 1). Spindles in the multifidus muscle were examined in six ways illustrated in Fig. 1. First, the multifidus muscle was divided into three segments along the mediolateral and dorsoventral axes (Fig. 2). The location of each spindle was recorded with respect to these segments. Second, the proximity of each spindle to a major fascial element was also examined. Each spindle within 500 μm of a connective tissue segment thicker than 100 μm was identified as being in “close proximity.” Third, the type, number and CSA of each intrafusal muscle fiber were determined for all spindles. Muscle spindles were classified as nuclear bag or chain on the basis of the relative diameter of intrafusal fibers. Based on the anatomy of spindles [10], those that contained both nuclear bag and chain intrafusal muscle fibers were classified as equatorial spindles, whereas those that contained only nuclear bag fibers were classified as polar spindles. Polar spindles were only analyzed for number and location as the area is highly variable depending where the section was made. Fourth, the CSA of the equatorial spindles was determined by drawing a line along the internal edge of the connective tissue capsule and calculating the contained CSA. Fifth, the thickness of the connective tissue capsule was quantified as the distance between the external edge of the capsule to the internal capsule at 8 equidistant positions around the spindle. Sixth, the proportion of muscle and connective tissue surrounding the spindles was determined. A region of interest (ROI) was set at twice the size of the average equatorial spindle CSA (calculated from all spindles in the present study; 10,500 μm^2^) and centered over the spindle. Muscle and connective tissue were individually selected and quantified using the threshold function in ImageJ (NIH, USA). Data are presented as a percentage of area positive for muscle or connective tissue in the ROI.

### Immunofluorescence assay and analysis

Slides were dewaxed and rehydrated using a series of three 2-min xylene and ethanol washes and blocked with 2% Bovine Serum Albumin in Tris-buffered saline (TBS). The sections were incubated in Ethylenediaminetetraacetic acid at pH 8.0 for 45 min at 90° Celsius. The sections were washed 3 × TBS before incubation overnight in Col I (1:200, AB6308, Abcam) and Col III (1:200, ab7778, Abcam) antibodies. Finally, sections were mounted with mounting media containing DAPI. No primary antibody controls were used. All slides were imaged using a AxioScan Z1 Scanner (Zeiss) and Collagen I and III staining intensity analysis was performed using ImageJ (NIH, USA). First, the mean grey value of 5 muscle fibers surrounding each spindle were averaged to determine the level of background staining. Next, the area and grey value of the pixels within the connective tissue capsule were determined. The pixels with grey values equal to or below the level of background staining were removed from the analysis. Mean grey value of the pixels within the capsule was determined and the staining intensity of Collagen I and III staining was calculated as the product of capsule area and mean grey value. Analysis was performed with the investigator blinded to group.

### Statistical analysis

Spindle location data were compared between groups and regions using a 2-way ANOVA analysis with a Sidak post hoc analysis. All remaining data were compared between IVD degeneration and control groups using t-tests. Significance was set at *P* < 0.05. All data are presented as mean ± SEM in text, tables and figures.

## Results

In total, 218 muscle spindles were identified. Of these, 117 were sectioned through the polar region of the spindle and were excluded from further measurement. The equatorial region was identified for 111 spindles. Spindles were either solitary (34.9%) or in groups of 2–6 spindles (65.1%).

### Muscle spindle number and location

**Fig. 3:**
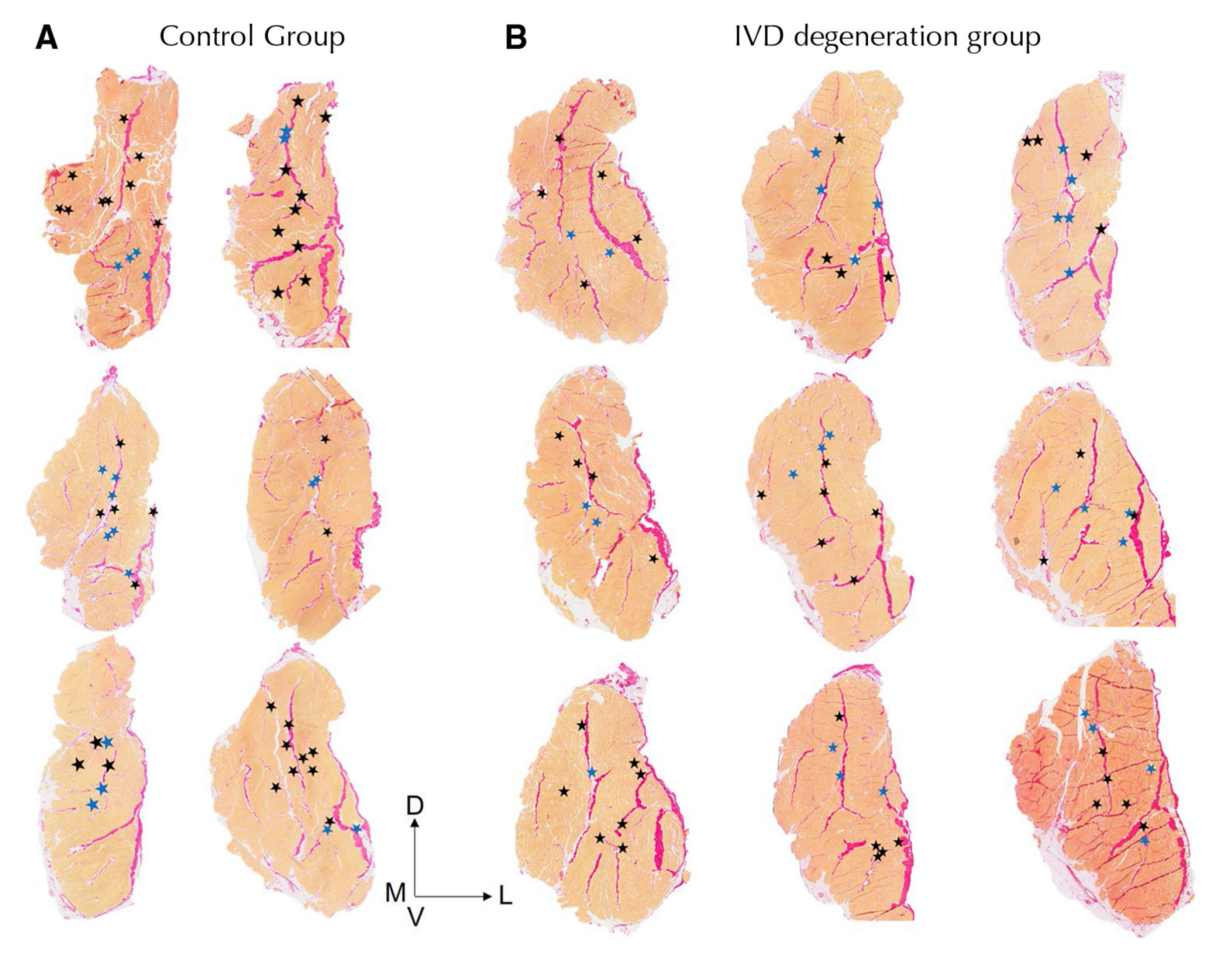
Location of muscle spindles in each muscle sample. Data are shown for all animals in the (**a**) control and (**b**) intervertebral disc (IVD) degeneration groups. Using Van Gieson’s stain connective tissue elements are pink and muscle is orange. D—dorsal; V—ventral; M—medial; L—lateral; Black star—single spindle; Blue star—compound spindle

**Fig. 4:**
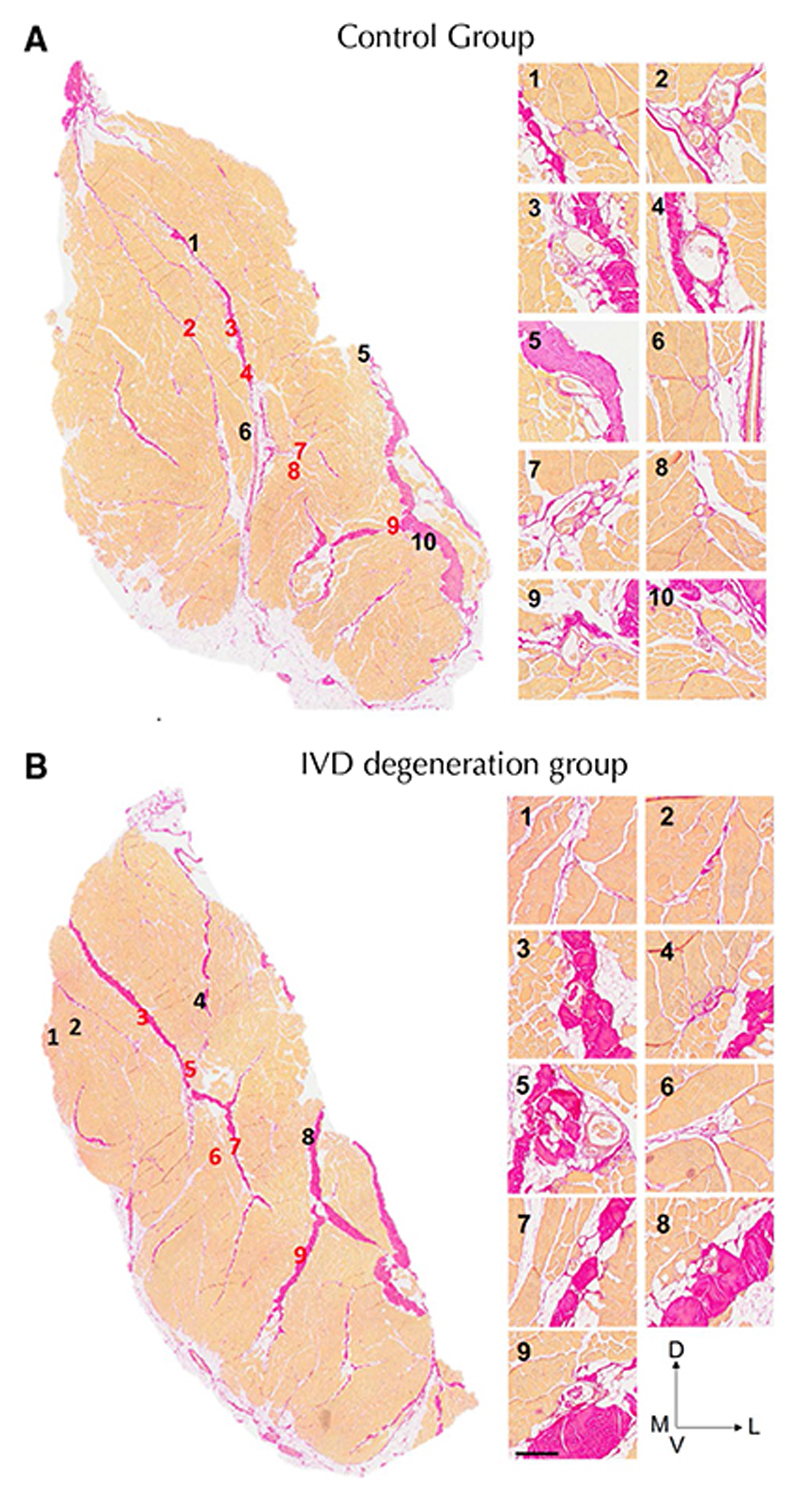
Location of muscles spindles relative to connective tissue for representative animals in the (**a**) control and (**b**) intervertebral disc (IVD) degeneration groups. Using Van Gieson’s stain connective tissue elements are pink and muscle is orange. The left panels show the location of each numbered spindle or cluster of spindles and the right panel shows each at higher magnification. Note the close proximity of most spindles to dense connective tissue in the muscle. D—dorsal; V—ventral; M—medial; L—lateral; IVD—intervertebral disc. Calibration—150 μm

Figures 2 and 3 show the location of muscle spindles by region and for each muscle sample, respectively. Spindles in the multifidus muscle are primarily located in the central third of the muscle along both the dorsoventral and mediolateral axes (Table 2). Of muscle spindles, 59.2% were in close proximity to a major fascial element in the muscle (see Fig. 4 for a representative sample from each group). No differences were detected in the number of spindles in the multifidus muscle or any feature of their location between the control and IVD degeneration groups (Table 2).

### Intrafusal fiber analysis

**Fig. 5:**
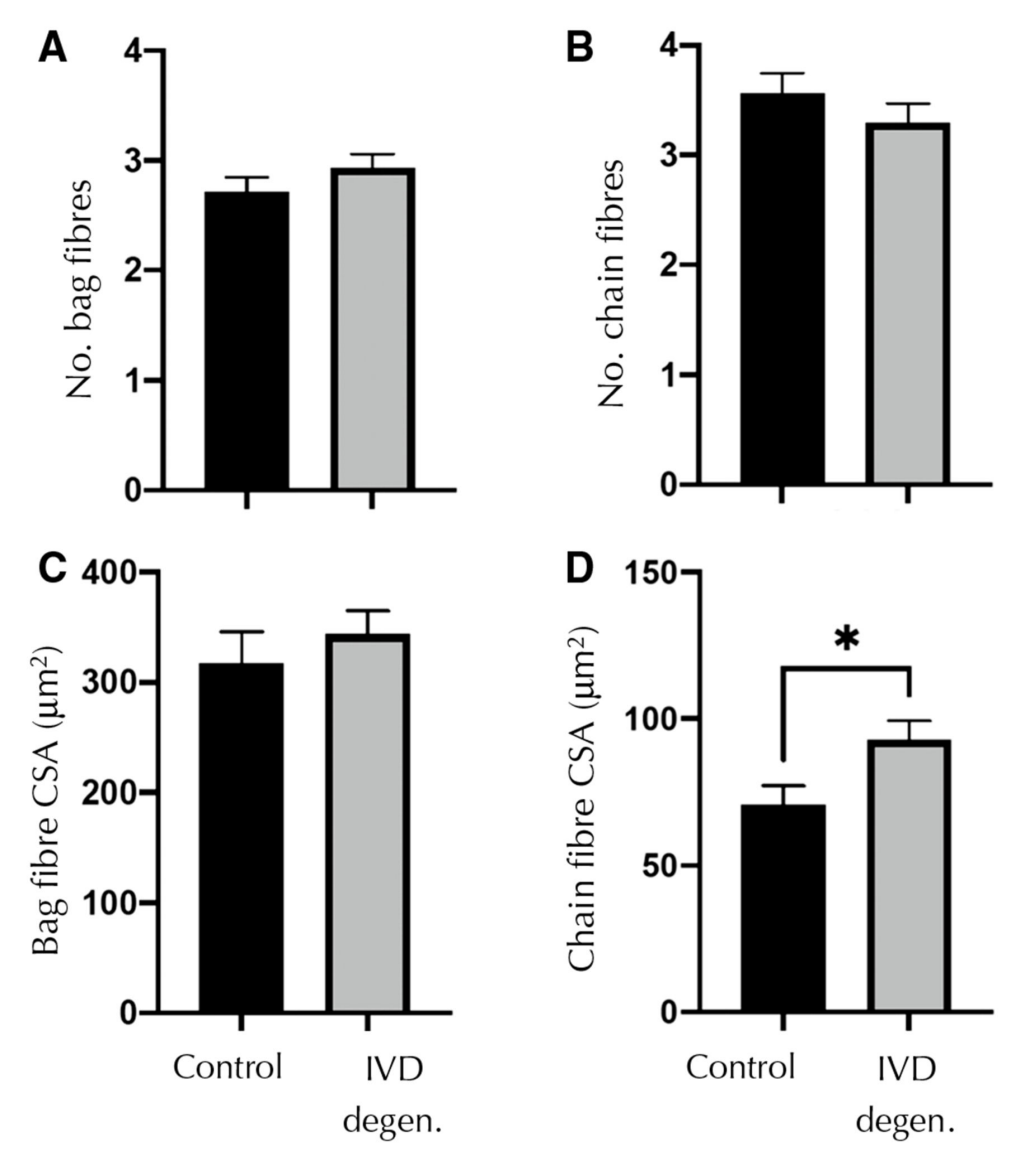
Number and cross-sectional area of intrafusal fibers for muscle spindles in control and (**b**) intervertebral disc degeneration (IVD degen.) groups. Data are shown for (**a**) and (**c**) nuclear bag and (**b**) and (**d**) nuclear chain fibers

Neither the number of nuclear bag fibers per spindle nor their CSAs were different between control and IVD degeneration groups in equatorial spindles in the multifidus muscle (Table 1; Fig. 5a and c). Although the number of nuclear chain fibers did not differ, their CSA was greater in the IVD degeneration group (Table 2; Fig. 5b and d).

### Muscle and connective tissue analysis

**Fig. 6:**
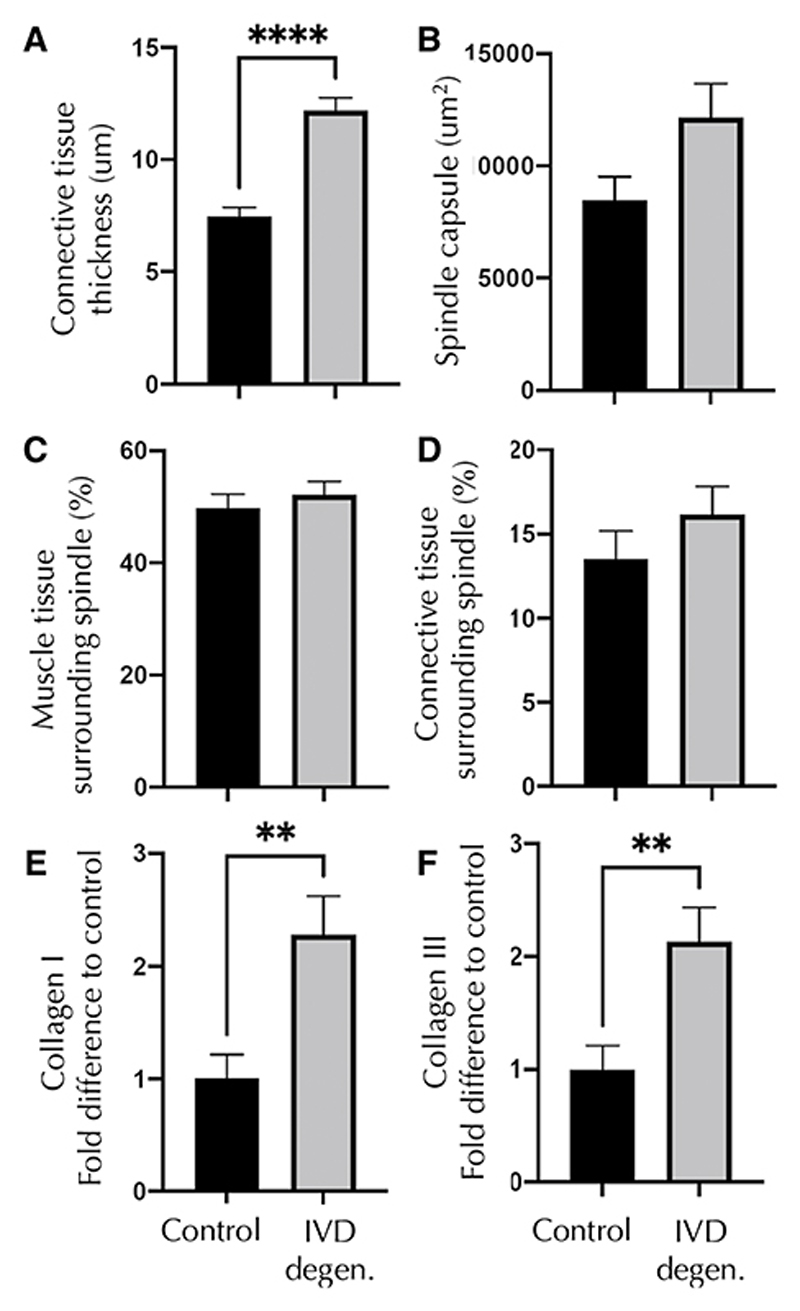
Connective tissue, capsule collagen and surrounding tissue measures. Data are shown for control and intervertebral disc (IVD) degeneration groups. **a** Connective tissue thickness averaged over measures and eight equidistant sites. **b** Muscle spindle capsule thickness. **c** Muscle tissue and **d** connective tissue proportion surrounding the muscle spindles. **e** Collagen I expression. **f** Collagen II expression. IVD—intervertebral disc

**Fig. 7:**
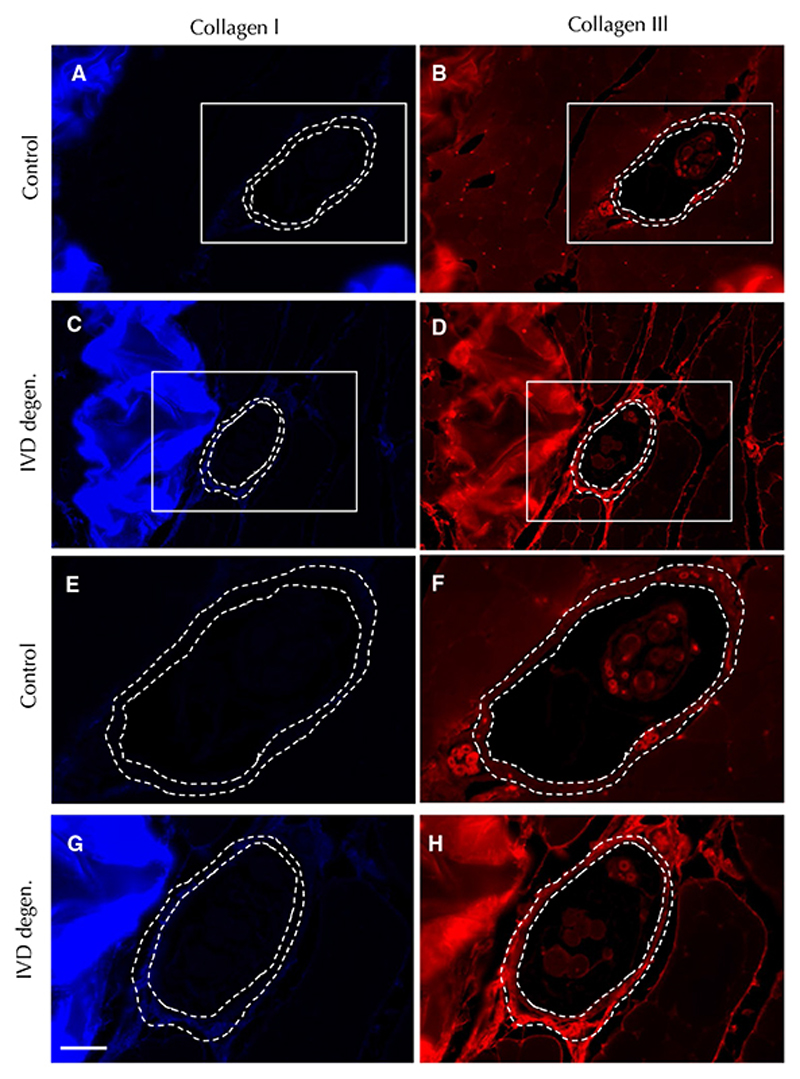
Collagen I (blue) and collagen III (red) staining of muscle spindle capsules. Images are shown for representative animals from the control (**a** and **b**) and intervertebral disc (IVD) degeneration (**c** and **d**) groups. The dashed lines delineate the inner and outer border of the connective tissue capsule. The boxed regions in (**a** to **d**) are magnified in (**e** to **h**) Calibration—50 μm

The thickness of the connective tissue layer surrounding the equatorial spindle capsules was greater in the multifidus muscle of animals with IVD degeneration than control animals (Table 2; Fig. 6a). IVD injury did not significantly change the proportion of muscle or connective tissue surrounding the spindles (Table 2; Fig. 6c and d). No differences were identified in the CSA of equatorial spindles (Table 2; Fig. 6b). The intensity of Collagen I and III staining was significantly greater in the connective tissue capsule of equatorial spindles in the IVD injury group than controls (Table 2; Fig. 6e and f, 7).

## Discussion

This is the first study to examine the potential impact of low back injury, specifically IVD injury, on morphology of muscle spindles in the multifidus muscle. The absence of differences in spindle number, location, CSA and intrafusal fiber number confirm that spindles do not undergo atrophy within six months after injury. Increased thickness of the connective tissue capsule and Collagen I and III expression provides evidence of fibrotic muscle spindle changes with IVD degeneration. Structural change to the muscle spindles would modify their mechanical properties, with potential to interfere with spindle sensory function. This novel observation provides a potential explanation for proprioceptive deficits and changes to motor control in IVD degeneration.

### Anatomy of multifidus muscle spindles

The anatomical location of muscle spindles has not been reported for the lumbar multifidus. Data are available for the cervical region of humans, which showed spindles primarily located in the anterolateral region of the multifidus muscle [23]. This differs from the location centrally along the dorsoventral and mediolateral axis of the lumbar muscle in sheep. It is not clear whether this is a region or species difference. Few muscle spindles are present in the most medial/ventral region of the multifidus which is the region that undergoes the most profound structural changes in models of IVD degeneration [2, 19].

Most muscle spindles were close to major areas of connective tissue. Although attachment of spindles to the perimysial fascia that surrounds bundles of muscle fibers is well known [24], their relationship to other fascial structures has not been described. This may have functional significance for sensation and control of distribution of tension [24]. Examination of the relationship between spindle location and multifidus proprioception requires investigation.

### Structural changes to muscle spindles with experimental IVD degeneration

The absence of signs of atrophy/loss of muscle spindles or intrafusal fibers in the multifidus in this model of IVD degeneration parallels animal models of muscular dystrophy. Those models induce substantial extrafusal fiber atrophy except in regions directly surrounding the spindles [25]. Preservation of spindles and surrounding extrafusal muscle has been interpreted to indicate a process of active sparing from degeneration [26]. We also report no obvious alterations to muscle and connective tissue in the region surrounding the spindles, despite the increase in connective tissue and muscle fibers changes in multifidus reported in this model [2]. In contrast to muscular dystrophy, aging induces intrafusal fiber atrophy, increased capsule CSA and spindle loss. Taken together this implies that muscle spindle changes observed in our injury model are not attributable to accelerated aging and imply an active process. Increased CSA of intrafusal fibers has been reported previously after issues such as denervation [27], which should not be present in out model, and requires further investigation. Whether atrophy of spindles and intrafusal fibers occurs in the chronic stage has not been studied.

### Impact of connective tissue changes on function

Alterations to the connective tissue capsule of multifidus muscle spindles are likely to impact their function. Increased spindle capsule thickness and increased Collagen I and III expression is similar to the fibrosis observed throughout the multifidus muscle (increased total connective tissue CSA [2] and epimysium (fascia that surrounds muscles) thickening [3]) in models of IVD degeneration. Fibrotic changes of the extrafusal multifidus muscle are thought to be promoted by inflammation [19, 21, 28]. Increased levels of pro-inflammatory cytokines are present in muscular dystrophy and aging [29], which also display spindle capsule thickening.

Thickening of the capsule has been considered as an adaptive, rather than maladaptive change, to protect the intrafusal muscle fibers [10]. Although plausible, fibrosis would also impact tissue mechanics. Muscle spindles are responsive to length change and rate of length change [10]. Although little data exists quantifying the relationship between capsule thickness and spindle function, modified stiffness of capsule would change deformation properties with length change. Previous work has highlighted the sensitivity of muscle spindles to even minor changes in stiffness of related structures, such as the thixotropic properties of muscle after contraction [30]. As Collagen I is a less elastic collagen subtype, its increased expression could impact the exposure of the sensory endings in the muscle spindle length changes, and thus their signalling of such events. Such modification of responsiveness of muscle spindles could underlie the proprioceptive deficits LBP. Modified afferent signals from the multifidus muscle have been identified as a primary mechanism for these proprioceptive defects [31, 32]. Direct evaluation of the impact of capsule collagen and thickness on spindle sensitivity is required.

### Limitations

As muscle spindles were examined in a single section multifidus for each animal it was not possible to quantify the location of all spindles in the muscle. Polar spindles were identified but were not included in detailed analysis of because of the large variation in CSA depending on the location of the section. It is important to consider that there are anatomical differences of the multifidus muscle between bipedal humans and quadrupedal sheep that suggest caution for translation of the present findings to humans. For instance, unlike human multifidus which has a large proportion of slow twitch muscle fibers [33], sheep multifidus muscle has predominately fast type muscle fibers [2, 19]. As some functional and structural properties of spindles differ between slow and fast muscles [34], this may impact the translation of these findings to humans. Functional changes in multifidus and proprioception were not evaluated in the sheep in this study, and so direct correlation with muscle spindle changes was not possible.

## Conclusion

This investigation has revealed that the muscle spindles undergo structural alterations in a model of IVD degeneration. The causes of these alterations and their impact on the multifidus muscle function and spine control in individuals with LBP requires further investigation.

## Figures and Tables

**Table 1:** Group means and statistical analysis of spindle location data between Control and IVD degeneration groups

	Control	IVD degeneration	Main effects *p*-value
*Dorsoventral axis*			G: 0.40
			**L: 0.005**
Dorsal third (%)	31.1 ± 7.3	26.3 ± 7.6	
Central third (%)	50.6 ± 9.4	52.5 ± 5.7	
Ventral third (%)	18.3 ± 9.5	21.2 ± 5.3	
*Mediolateral axis*			G: 0.55
			**L: < 0.001**
Medial third (%)	5.0 ± 2.6	3.7 ± 1.5	
Central third (%)	65.9 ± 9.6	68.3 ± 5.0	
Lateral third (%)	29.0 ± 8.8	28.1 ± 5.5	

Values in bold are statistically significant

*G*, group; *L*, location

**Table 2:** Group means and statistical analysis of spindle data between Control and IVD degeneration groups

	Control	IVD degeneration	*p*-values
Total spindle number (n)	14.7 ± 3.1	14.4 ± 1.1	0.94
Spindles close to connective tissue (%)	58.8 ± 6.9	59.4 ± 6.0	0.95
Number of bag fibers (n)	2.7 ± 0.1	2.9 ± 0.1	0.25
Number of chain fibers (n)	3.6 ± 0.2	3.3 ± 0.2	0.31
Bag fiber CSA (μm^2^)	316.7 ± 29.0	343.6 ± 21.6	0.45
Chain fiber CSA (μm^2^)	70.8 ± 6.4	92.8 ± 6.5	**0.020**
Spindle capsule CSA (μm^2^)	8461 ± 1054	12,151 ± 1521	0.061
Capsule connective tissue thickness (μm)	7.5 ± 0.4	12.2 ± 0.6	**< 0.001**
Muscle tissue surrounding spindles (%)	47 ± 3.1	52.2 ± 2.3	0.48
Connective tissue surrounding spindles (%)	13.5 ± 1.6	16.2 ± 1.7	0.28
Collagen I staining intensity (fold difference)	1 ± 0.3	2.3 ± 0.3	**0.005**
Collagen III staining intensity (fold difference)	1 ± 0.2	2.1 ± 0.4	**0.006**

Values in bold are statistically significant

*CSA*, cross-sectional area; *IVD*, intervertebral disk

## Data Availability

The datasets generated during and/or analyzed during the current study are available from the corresponding author on reasonable request.
